# Parental Psychological Control and Emotional and Behavioral Disorders among Spanish Adolescents

**DOI:** 10.3390/ijerph16030507

**Published:** 2019-02-12

**Authors:** Benito León-del-Barco, Santiago Mendo-Lázaro, María I. Polo-del-Río, Víctor M. López-Ramos

**Affiliations:** Department of Psychology, Faculty of Teacher Training College, University of Extremadura, 10071 Caceres, Spain; mabelpdrio@unex.es (M.I.P.-d.-R.); vmlopez@unex.es (V.M.L.-R.)

**Keywords:** mental health, emotional disorders, behavioral disorders, adolescents, family, parental psychological control, parents

## Abstract

There is no denying the fundamental role played by parents in the psychosocial development of their children—either as a liability or as protection against mental health disorders. This study seeks to ascertain, by means of odds ratio statistics (OR), the correlation between parental psychological control and emotional and behavioral disorders. A total of 762 students took part in this study, with an average age of 12.23 years—53.8% of whom were girls and 46.2% were boys. Children and adolescents reported their parental psychological control and their emotional and behavioral disorders (i.e., emotional and behavioral problems, internalizing and externalizing problems). Minors who perceive their psychological control as high are 6 times more likely to suffer from internalizing disorders and 4.8 times more likely to develop externalizing disorders. Furthermore, the probability of suffering externalizing disorders is higher among males who perceive a high degree of psychological control. This study breaks new ground on the importance of perceived psychological control—considered as a negative form of control by parents—in the emotional and behavioral disorders among children and adolescents.

## 1. Introduction

### 1.1. Parental Psychological Control

Parents constitute the main means of development and socialization for most people from a very early age. By the same token, the role of the family unit in psychosocial development is undeniable, with parents being the most powerful force in their children’s lives. Differences in parenting styles have been used to account for the effects of familial socialization on children’s social competence; these styles and their differences result from the interaction of different attitudes and behaviors displayed by parents toward their children [1], and they have a direct influence on children’s behavior, emotional security, and well-being [2,3].

More specifically, the work of Baumrind [4] is of particular relevance in the study of parenting styles, since the author outlined a range of parenting styles that are widely accepted in the relevant literature. Still, a major limitation of research on parenting styles is that parents do not follow a predefined pattern. Indeed, different parenting styles are not mutually exclusive, as parents may abide by a specific style by and large together with specific parental practices that are usually associated with a different one. In this regard, most research follows Maccoby and Martin’s [5] two-dimensional framework, classifying parents into four styles according to two dimensions: Affect and control.

It is rather uncommon for research on parenting styles to focus solely on one of these dimensions: Either affect or control. About affect, Rohner [6] claims that parental acceptance—rejection constitutes a continuum, with parents who express love and affection (both verbally and physically) for their children lying at one end. At the other end, one finds parents who feel aversion for their children, criticize them, and reject them. Affect and communication prevent disorderly conducts among adolescents and stimulate a positive development [7,8]. Parents’ positive affect towards their children reveals itself as a preventive factor for mental health [9]: Children raised in a warm and affectionate environment are less likely to develop behavioral problems than children raised in environments lacking adequate affection. On a related note, research [10,11,12] corroborates that inadequate levels of affect and support, and the prevalence of aggression and rejection toward children are linked to the manifestation of behavioral disorders, like aggressiveness, hostility, and crime.

With regards to the dimension of control, and despite the fact that there exists research that underscores the importance of parental control in the development and autonomy of the child [13,14], it remains a controversial dimension because of its complexity as a construct since, even though there is a consensus about the negative association between parental control and behavioral problems [13,15,16], the specific components of parental control that contribute to preventing emotional and behavioral disorders are often not clear. Indeed, results from different studies on the effects of parental control on children are often contradictory [17,18]. Adolescents whose parents are permissive and indulgent present behavioral problems less frequently than adolescents whose parents use authoritarian or negligent styles [19,20], and emotional problems are frequent among children of authoritarian parents [21].

Thus, Barber [15] distinguishes between behavioral control and psychological control, in an attempt to clarify the construct of parental control from a conceptual perspective. Behavioral control refers to parents’ efforts to regulate and monitor the child’s behavior, and it is considered a buffering factor for children’s emotional distress and behavioral problems when such parental supervision is appropriate to the child’s age and is provided along with respect for autonomous development, emotional warmth, and communication [22,23].

On the other hand, psychological control is associated with emotional problems [24]; the term refers to manipulation strategies used by parents on their children, such as emotional blackmail, guilt induction, overwhelming impositions, or love withdrawal, and research has found such strategies to be opposed to autonomy granting [25,26]. In fact, parents who display few autonomy granting behaviors tend to use psychological control strategies when adolescents act in ways that they disapprove of [27]. 

Psychological control has traditionally been considered a negative form of control in that it affects the child emotionally, stifling their ability to establish emotional links with others, the development of their personal identity, and their autonomy [15]. Research on the consequences of psychological control on children’s adjustment reveals a higher risk of having internalizing problems. This research also shows that psychological control is positively correlated with depressive symptoms [18,28,29,30], low self-esteem, and low self-concept [24,31,32], as well as problems with emotional regulation [1,2,3,33].

### 1.2. Emotional and Behavioral Problems

The analysis of mental health among children and adolescents who may be at risk of emotional or behavioral disorders has become a major focal point for researchers on a global scale, and it is now a priority for public health policies. Emotional and behavioral problems among children and adolescents indeed attract much social concern in that they are associated with disability, suffering, functional deterioration—not to mention the economic cost for public health services worldwide [34]. It is estimated that between 10% and 20% of the world’s minors suffer some sort of mental health problem [35]. Emotional and behavioral symptoms are frequent during adolescence because the changes and adaptation processes people face during this stage of development can be emotionally distressing and expressed as behavioral problems [36], affecting personal well-being and relationships with other people. In this context, the family’s flexibility to adapt itself to the changes required by the adolescent, as well as the quality of communication among family members and their emotional bonds, have effects on the adolescent’s degree of vulnerability [37].

Therefore, the family unit can be a key protective factor in preventing mental health problems or disorders [38]. Parents’ conceptions about upbringing, interpersonal relationships, and their own upbringing styles are determining factors in the development of the child. It is thus paramount to understand individual differences in the relationships between parents and adolescents, as the development of the latter is shaped by the quality of such relationships [39]. 

Research on the association between parenting styles and psychopathology during adolescence has shown that, when parent—child relationships are characterized by a warm family climate, where communication is possible and rules are established and enforced using flexibility, adolescents tend to be well adjusted and adaptable. On the contrary, when affection and communication are lacking, family rules are rigid, and parents have a negative perception of their children, conflicts in the parent—child relationship can have adverse effects on adolescents’ personal and socioemotional development [40]. Clear associations between internalizing problems (passivity, apathy, depressed mood, and psychological distress, among other manifestations) and an authoritative parenting style have been reported [41]. Inadequate levels of affection and the predominance of aggression and rejection toward children are associated with the expression of behavioral problems of aggressiveness, hostility, and delinquency [10,11]. It has also been suggested that insufficient affection received from the mother combined with rejection by the father is associated with a higher probability for the child to play the role of the bully in school settings [42].

### 1.3. This Study

The purpose of this study is, primarily, to determine the link between parental psychological control and mental health (emotional and behavioral problems) by means of odds ratio statistics (OR). We hypothesize that a high degree of psychological control—exerted in the form of manipulation strategies, such as emotional blackmail and guilt induction—is a risk factor for the presence of emotional and behavioral problems. To begin with, to analyze the role of gender and age in the variables that are the object of study here, multivariate comparisons are drawn between the average score for psychological control and the prevalence of emotional and behavioral problems according to gender and age/school level (≤12 or ≥13). It should be noted that, on the one hand, research on the effects of psychological control according to gender and age are inconsistent. Some research suggests it has a more negative effect on girls [43], whereas some others report similar findings about boys [44,45]. As far as age is concerned, psychological control is more harmful during developmental periods of high autonomy [46]. About mental health (behavioral and emotional problems), most research indicates that boys score higher on externalizing symptoms, such as behavioral problems and hyperactivity, whereas girls score higher on internalizing symptoms, such as emotional problems [47,48,49,50,51]. By contrast, international research, such as the Spanish studies cited here, is inconclusive with regards to age.

The variables that are the object of study here have been assessed by means of a self-report. Why are we interested in the perception children have about the type of control their parents exert? There is a widespread mismatch between parents’ and children’s opinions about parental practices. Parents’ usually hold a skewed view about their own practices, often as a result of a social desirability bias. By contrast, the perception of adolescents is not as skewed; it is, therefore, more objective, and it can be a reliable predictor of their responses—much more so than in the case of parents [22].

Thus, we used the self-report version of the Strengths and Difficulties Questionnaire (SDQ) to evaluate emotional and behavioral problems. This questionnaire has proven an excellent screening tool when compared to earlier ones, like the Child Behavior Checklist [51,52]. It has also received international validation, and it has been used in many international studies for the evaluation of mental health [53]. The analysis of the psychometric properties of the Spanish version of the self-report version of the SDQ seems to corroborate the notion that it is a useful and suitable tool for the detection of emotional and behavioral problems among adolescents [49].

## 2. Materials and Methods

### 2.1. Sample

The study sample was selected through a conglomerate, multi-age sampling plus the random selection of school classes in schools with more than one group for 5th and 6th grade (primary school) and the 1st and 2nd year of secondary school. The conglomerate sampling was carried out after randomly selecting four schools. In terms of the distribution by school year of the participants, 22% of them were studying the 5th grade; 23.1% the 6th grade; 26.3% the 1st year of secondary school; and 28.6% the second year of secondary school. The size of the sample of participants was determined in relation to the aggregate number of primary and compulsory secondary education students in public and subsidized schools in Extremadura (Spain) during the 2017–2018 academic year. We considered a sampling error of 3 percentage points and a reliability level of 95.5%. The sample of participants comprised 762 students in total. The average age was 12.23 years (SD = 1.122; range 11–14); 53.8% females (*n* = 410) and 46.2% (*n* = 352) males. In Spain, the transition from primary education to secondary education (between 12 and 13 years of age) is considered as a critical stage because it coincides with the first steps toward autonomy, especially autonomy from the family [54]; therefore, controlling for age is especially relevant during this transition period that takes place at the beginning of adolescence.

### 2.2. Data Collection Instruments

#### 2.2.1. Strengths and Difficulties Questionnaire, SDQ

The self-report version of the SDQ [54] is a concise instrument, which reveals itself as an excellent screening tool for mental health disorders in minors. The internal consistency of all its scales has been highlighted both at an international level [53,55] and in its Spanish version [49,56]. The questionnaire comprises 25 items, which are divided into 5 scales: 1. Emotional symptoms (e.g., I have many fears, I am easily scared), 2. Conduct problems (e.g., I get very angry and often lose my temper), 3. Peer relationship problems (e.g., I am usually on my own. I generally play alone or keep to myself), 4. Hyperactivity/inattention (e.g., I am restless, I cannot stay still for long), 5. Prosocial behavior. Each scale is assessed through five items, and responses to these are based on a three-point Likert-type scale (where 0 indicates responses of “not true,” 1 “somewhat or sometimes true,” and 2 “very true or often true”). For group samples, it is advisable to put together the items of the conduct problems and hyperactivity/inattention scales into a new scale called externalizing problems [57]. By the same token, the items in the emotional symptoms and peer relationship problems are also combined into the scale of internalizing problems [58]. 

Both the aggregate score of the SDQ and the separate scores for each scale are classified into three broad categories: ‘Normal’, ‘subclinical’, or ‘clinical’. In the original score scale [52], the boundary of the clinical category corresponds to the top 10% of cases (percentile ≥ 90), whereas the subclinical category accounts for the cases between the 80th and 90th percentiles. The global score for difficulties is calculated by adding up the four scales—prosocial behavior not being considered. The global reliability of the scale yields a Cronbach’s alpha (α) of 0.75 and a compound reliability (CR) of 0.80; while the externalizing problems scale has an α of 0.71 and a CR of 0.76. In turn, the internalizing problems scale yields an α of 0.73 and a CR of 0.72.

#### 2.2.2. Scale for the Evaluation of the Educational Style of Adolescents’ Parents, Children’s Version (EES-C)

EES-C [22] comprises 41 items grouped into six scales (affect—communication, autonomy development, behavioral control, psychological control, revelation, and humor), whose responses also follow a Likert-type scale with six degrees of agreement (1–6) covering a continuum that ranges from ‘Completely Disagree’ to ‘Completely Agree’. The present study has only focused on the factor of psychological control (8 items), which evaluates children’s perception of their parent’s use of manipulation strategies, such as emotional blackmail and guilt induction: “They won’t talk to me when they are mad at me,” “they make me feel guilty when I don’t do as they say,” “they give me the cold shoulder if I do something they don’t like.” It has an α of 0.82 and a CR of 0.83.

### 2.3. Procedure

This study was approved by the Bioethics and Biosafety Committee of the University of Extremadura (No. 0063/2018). The ethical principles and code of conduct of the American Psychological Association (2010) have been followed with regards to the informed consent that parents had to sign because of the age of the participants. First, we contacted the schools to explain the goals of this research and to ask for the necessary permission to have the questionnaires filled out by their students. Data were collected by administering the forms to each class in the presence of a trained researcher. In addition, anonymity and confidentiality were ensured for all participants, while we made sure that responses and all other data were to be used for research purposes exclusively. The questionnaires were administered during the school day, usually taking around 25 min—it was filled out in a suitable environment, free from distractions.

### 2.4. Data Analysis

Prior to the statistical analysis, a missing data analysis was performed with the variables included in the models under study. These statistical analyses were carried out with the SPSS suite, PC v. 21.0, and they consisted of an instrument reliability analysis, a multifactor variance analysis, an odds ratio association measure and, lastly, to identify groups, establish the relationships among groups, and predict future events, a classification model was created through a decision tree method—more specifically, CHAID (chi-square automatic interaction detector) was utilized. 

## 3. Results

### 3.1. The Role of Gender and Age in the Variables under Study: Multiple Factor Analysis

To assess the effect of gender and age on the variables that were the object of study, we carried out a comparative multivariate analysis of the mean scores for the whole SDQ (emotional and behavioral problems), for the internalizing problems scale, for the externalizing problems scale, and for the score of the psychological control factor of the EES-C based on gender, age (≤12 or ≥13), and the interaction between both variables (Table 1).

The multivariate analysis (MANOVA) revealed a major influence of gender (Wilks λ = 0.951, F(3, 754) = 13.049, *p* < 0.001, ƞ^2^ = 0.049), age (Wilks Λ = 0.977, F(3, 754) = 5.980, *p* < 0.001, ƞ^2^ = 0.023) and the interaction between gender and age (Wilks Λ = 0.984, F(3, 754) = 4.005, *p* = 0.008, ƞ^2^ = 0.016).

With regard to the variable of psychological control, univariate comparisons indicate that boys score higher than girls (F(1, 756)= 19.345, *p* < 0.001, ƞ^2^ = 0.025). There are no significant differences by age or by the interaction of gender and age. As far as the global score and the scales of the SDQ are concerned, univariate analysis shows that boys score higher on the variable of emotional and behavioral problems (F(1, 756) = 8.132, *p* = 0.004, ƞ^2^ = 0.011) and on the variable of externalizing problems (F(1, 756) = 23.24, *p* < 0.001, ƞ^2^ = 0.030). Participants aged 12 or younger, in turn, score higher on the variable of emotional and behavioral problems (F(1, 756) = 12.691, *p* < 0.001, ƞ^2^ = 0.017), the variable of externalizing problems (F(1, 756) = 4.619, *p* = 0.032 ƞ^2^ = 0.006), and the variable of internalizing problems (F(1, 756) = 14.578, *p* < 0.001, ƞ^2^ = 0.019). Again, there is no interaction between gender and age.

### 3.2. Psychological Control and Emotional and Behavioral Problems: Odds Ratio (OR) Association Measure

Our goal was to ascertain the correlation between parental psychological control and mental health by means of OR statistical tools. To do so, the dependent variables utilized here were the global score for the SDQ (emotional and behavioral problems), the score on the scale of internalizing problems, and the score on the scale of externalizing problems. These variables were treated as dichotomous variables, with the dividing line set on the 80th percentile; i.e., P ≥ 80 = Having problems; P < 80 = Not having problems. On a further methodological note, the participants from the clinical and subclinical categories in the original scale [50] were integrated into the category “Having Emotional and Behavioral Problems, Having Internalizing Problems, and Having Externalizing Problems.” The independent variable in this study was the psychological control factor of the EES-C—once again taken as a dichotomous variable (Normal = 0; High Psychological Control = 1) with the boundary set at the 80th percentile (P < 80 = 0; P ≥ 80 =1).

As can be observed in Table 2, there is a significant positive correlation between the variable of psychological control and the variables of emotional and behavioral problems, internalizing problems and externalizing problems. More specifically, regarding the variable of emotional and behavioral problems, the odds ratio (OR = 5.986), the limits of the ratios at 95% confidence, and the χ^2^ test (= 95.099) corroborate the significance of these results (*p* < 0.001). Likewise, in relation to the variables of internalizing problems and externalizing problems, both the limits of the ratios and the χ^2^ test demonstrate how significant the results are (*p* < 0.001).

Thus, the OR tests suggest that (1) belonging to the group of children whose perceived psychological control is high (through manipulative strategies, such as emotional blackmail or guilt induction) is a risk factor for emotional and behavioral problems with respect to children who report normal or low psychological control, and the likelihood of having mental health problems is 5.986 times greater in the group where perceived psychological control is high than in the group with normal psychological control; (2) the likelihood of having internalizing problems is 3.035 times higher in the group where perceived psychological control is high, and (3) the chance of having externalizing problems is also 4.804 times higher in the high psychological control group.

### 3.3. Interpreting the Correlations and the Role of Gender and Age: Classification Tree

**Figure 1:**
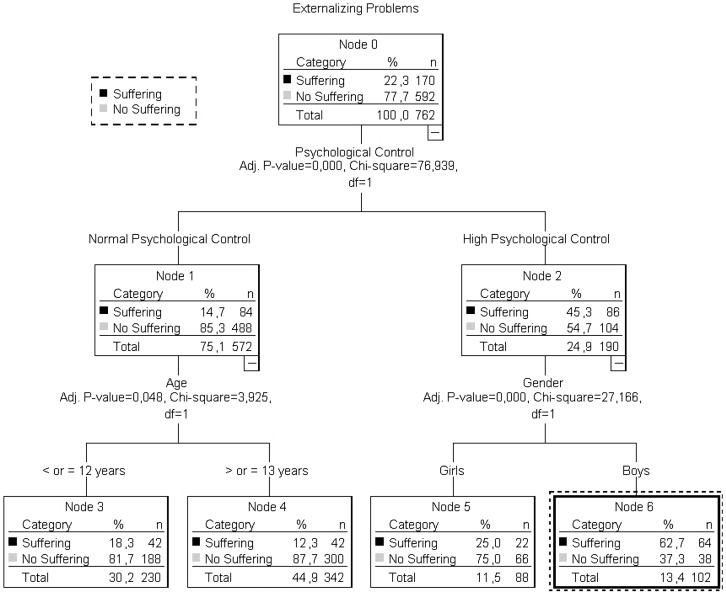
Classification tree for externalizing problems.

Lastly, to clarify the interpretation of the correlations outlined above and the role of gender and age in the present study, we performed a classification tree analysis for each dependent variable (emotional and behavioral problems, internalizing problems and externalizing problems) while introducing psychological control and the different gender and age groups as independent variables. In the case of emotional and behavioral problems and internalizing problems, the role of age and gender does not seem to bear any significance among participants who perceive high psychological control. However, the classification tree (Figure 1) for the variable of externalizing problems shows some correlation with gender among those participants whose perceived psychological control is high. 

The accuracy of the classification tree lies at 81.1% (Risk = 0.189; SE = 0.014). We can observe in Figure 1 that the lowest probability of presenting externalizing problems (11.3%) corresponds to participants under 13 years of age who perceive low and average psychological control (Node 4), whereas the highest probability of externalizing problems (62.7%) is to be found among males who perceive high psychological control (Node 6).

## 4. Discussion

Regarding our main research goal, i.e., establishing the correlation between parental psychological control and mental health (emotional and behavioral problems), the results from the OR test indicate that, as a general rule, parental psychological control is a risk factor for both internalizing and externalizing emotional and behavioral problems. In this regard, our research into the connection between these variables has singled out psychological control as one of the leading factors underlying emotional disorders among minors [15,59]—both internalizing [18,28,29,30] and externalizing [60]. 

Research suggests that psychological control correlates more positively with internalizing problems (e.g., [18,24,61]) whereas behavioral control correlates more positively with externalizing problems [60,62]. However, in our study, based on subjects who perceive high psychological control, the likelihood of externalizing problems is notably higher than that of internalizing problems. This overlaps to a large degree with results from a clinical sample—utilizing the same tools to measure psychological control (EES-C)—which concludes that adolescents score higher on externalizing symptoms and a higher risk of externalizing symptoms, associated to psychological control [59]. These similarities may be due to the criteria that were used to identify participants who had emotional and behavioral problems in their teenage years [52], which would confirm the usefulness of the SDQ scale as a tool for the detection of mental health problems [49,51,52]. 

In addition to the above, there is no denying the connection between children’s psychological adjustment and parents’ behavior. Indeed, the effect of the conduct of adolescents on the changes in children’s upbringing has been reported widely [63,64,65,66,67]. Externalizing problems are manifested in behaviors, like aggressiveness, hostility, disobedience, and crime [68]. Some parents strive to control these conducts by resorting to manipulative strategies; e.g., by using emotional blackmail or rejection as a way of disciplining their children [5,69].

Last, regarding the role of gender and age in the connections discussed here, there is only one relevant case. Namely, the classification tree for the variable of externalizing problems shows the significance of gender in the group of participants who perceive high psychological control. More specifically, the participants who are most likely to have externalizing problems (62.7%) are males whose perceived psychological control is high. These results corroborate research indicating that the negative effects of psychological control are more severe in boys than in girls [44,45]. It is generally believed that parents still behave more strictly and rigidly with their sons than with their daughters [70]. Several examples of research done in Spain support the idea that boys perceive their parents with a higher degree of rejection [71,72,73].

Similarly, boys score higher on the variables of emotional and behavioral problems and externalizing problems. By and large, it can be argued that being a boy is a risk factor for the prevalence of mental health problems, as opposed to being a girl. These results are in line with most research on the subject that, regardless of the version of SDQ utilized (parents, teachers, self-report), confirms that boys score higher on externalizing symptoms, such as conduct problems or hyperactivity [47,48,50].

### Study Limitations and Future Directions 

The present study has several limitations, most notably the use of self-reports to assess both mental health problems and psychological control. We believe it would be necessary to rely on other informants in addition to participants themselves. On a related note, within the framework of a bidirectional model for the relationship between parents and children, it may be necessary to assess mental health and psychological control from the point of view of parents. In addition, despite paternal and maternal styles overlapping to a great extent [22,74], using one self-report for both parents limits the possibility of establishing and monitoring differences between them, if any do exist. 

Furthermore, the ethnic and cultural context of our study population must be considered for further interpretation and generalization of the results presented in this paper. Several studies carried out in Spain and other European countries, as well as in Latin America [18,26,75,76,77,78,79,80], suggest that the association between parenting styles and the emergence of clinical conditions varies according to the cultural context. Thus, the influence of culture implies that these results cannot be extrapolated to other countries, especially outside the Western world [81].

## 5. Conclusions

The conclusions that can be drawn from this study confirm that the likelihood of having emotional and behavioral problems is 5.986 higher for the group with high psychological control, as opposed to that of participants who perceive normal psychological control. In line with this, the group with high perceived psychological control is 3.035 times more likely to have internalizing problems and 4.804 times more likely to have externalizing problems. More specifically, it is the male participants who perceive high psychological control who are the most likely to suffer externalizing problems.

The analysis of mental health risks related to emotional or behavioral disorders in children and adolescents is a key research area worldwide, and it has become a major concern for public health policy-makers. We are, of course, aware of how emotional and behavioral problems in children and adolescents have given rise to much social concern: Not only are they linked to disabilities, suffering, and functional impairment, but they also represent a substantial economic burden for healthcare systems on a global scale. This study makes a significant contribution toward understanding the importance of parents’ psychological control in the emotional and behavioral problems of children and adolescents. The family unit can be a key risk factor (or a protective one) regarding mental health problems. Psychological control is considered a harmful element in parent—child relations: The use of strategies, such as emotional blackmail, guilt induction, love withdrawal, etc., may constitute intrusive behaviors verging on psychological abuse. 

Finally, we believe in the value of intervention and training programs for families. Preventive intervention programs for parents can help to develop protective mechanisms for children’s development. A remarkable example is the ACT—Raising Safe Kids Parenting Program, developed by the American Psychological Association, (APA, Washington, EE. UU.), which has been applied in different countries (the USA, Japan, Peru, Colombia, Greece, Bosnia, Turkey, Taiwan, Brazil, and Portugal) with the purpose of developing parenting skills and knowledge through the dissemination of non-violent discipline approaches, anger management techniques, conflict resolution skills, as well as information about child development and the effects of communications media [82].

Training parents is part and parcel of children’s upbringing. In addition, this method promotes their development, facilitates the learning of tools for conflict resolution, improves parent—child relations, provides patterns for assertive, effective communication, and heightens the sense of satisfaction and competence among parents when dealing with their parental duties and responsibilities. We also believe in a type of parental training that allows parents to realize that the source of their children’s problems may lie within the parents themselves. 

Interventions focused on parents would be of a cognitive behavioral nature, their goals being to improve emotional communication skills (active listening), discipline-oriented communication (establishing clear instructions, boundaries, and rules, as well as behavioral expectations and consequences), discipline and behavior management skills (how to use specific reinforcers and solve problems related to children’s behaviors), and prosocial skills development (teaching parents how to raise their children to share and cooperate). The time allocated to these programs and their intensity should be planned with the aim of producing lasting results, and even though the proposed intervention is interactive and parents are provided with information, facilitators should use skills and training techniques based on observational learning. Among the possible techniques of this sort are, for instance, the presentation of live or virtual models representing the role of parents for helping trainees to learn parenting skills, conduct rehearsals with trainers and with their children, and tasks to be carried out at home.

An important insight parents should acquire is that parental control is necessary during infancy to help children to organize and guide their behavior and regulate their emotions, and then, parental control decreases as the adolescent develops as an autonomous person capable of regulating their emotions. This letting go of control is one of the most important adjustments parents must make to accommodate the new needs of their adolescent children and stimulate their autonomous development [39].

## Figures and Tables

**Table 1:** Descriptors for the variables of the study: Emotional and behavioral problems, internalizing problems, externalizing problems, and psychological control by age and gender groups.

Variables	Gender	Age ≤ 12 years		Age ≥ 13 Years		Total	
		*M*	*SD*	*M*	*SD*	*M*	*SD*
Emotional and Behavioral Problems	Girls	16.09	4.52	14.84	3.99	15.28	4.22
	Boys	17.04	5.59	15.85	4.63	16.40	5.13
	Total	16.60	5.13	15.26	4.29	15.80	4.69
Internalizing Problems	Girls	8.01	2.93	6.81	2.78	7.24	2.89
	Boys	7.65	3.07	7.24	2.61	7.43	2.84
	Total	7.82	3.01	6.99	2.72	7.33	2.86
Externalizing Problems	Girls	8.08	2.67	8.02	2.18	8.04	2.36
	Boys	9.39	3.18	8.60	2.66	8.97	2.94
	Total	8.77	3.02	8.26	2.41	8.47	2.68
Psychological Control	Girls	19.22	7.99	19.33	8.18	19.28	8.10
	Boys	22.07	9.44	22.08	8.77	22.07	9.08
	Total	20.73	8.89	20.48	8.54	20.58	8.68

*M* = mean, *SD* = standard deviation.

**Table 2:** Odds ratio according to the dependent variables under study (emotional and behavioral problems, internalizing problems, externalizing problems) and psychological control.

SDQ Problems	Psychological Control	% Problems	Rate	χ2	*p*	OR	95% CI	
Emotional and Behavioral	Normal	11.8	0.447	95.099	<0.001	5.986	4.081	8.781
	High	44.7	0.553					
Internalizing	Normal	15.7	0.570	35.884	<0.001	3.035	2.090	4.407
	High	36.2	0.430					
Externalizing	Normal	14.6	0.494	76.939	<0.001	4.804	3.326	6.939
	High	45.3	0.506					

Rate = Ratio of the categories for psychological control where problems were detected. OR: odds ratio. CI: confidence interval.
